# The Antimicrobial Peptide Lysozyme Is Induced after Multiple Trauma

**DOI:** 10.1155/2014/303106

**Published:** 2014-08-31

**Authors:** Tim Klüter, Stefanie Fitschen-Oestern, Sebastian Lippross, Matthias Weuster, Rolf Mentlein, Nadine Steubesand, Claudia Neunaber, Frank Hildebrand, Thomas Pufe, Mersedeh Tohidnezhad, Andreas Beyer, Andreas Seekamp, Deike Varoga

**Affiliations:** ^1^Department of Trauma Surgery, University of Kiel, Arnold-Heller-Straße 3, 24105 Kiel, Germany; ^2^Department of Anatomy, University of Kiel, Otto-Hahn-Platz 8, 24118 Kiel, Germany; ^3^Department of Trauma, Hannover Medical School, Carl-Neuberg-Straße 1, 30625 Hannover, Germany; ^4^Department of Trauma Surgery, University of Aachen, Pauwelsstraße 30, 52074 Aachen, Germany; ^5^Department of Anatomy and Cell Biology, RWTH Aachen University, Wendlingweg 2, 52074 Aachen, Germany; ^6^Department of Cardiovascular Surgery, University Medical Center of Schleswig-Holstein, Kiel Campus, Arnold-Heller-Straße 3, 24105 Kiel, Germany

## Abstract

The antimicrobial peptide lysozyme is an important factor of innate immunity and exerts high potential of antibacterial activity. In the present study we evaluated the lysozyme expression in serum of multiple injured patients and subsequently analyzed their possible sources and signaling pathways. Expression of lysozyme was examined in blood samples of multiple trauma patients from the day of trauma until 14 days after trauma by ELISA. To investigate major sources of lysozyme, its expression and regulation in serum samples, different blood cells, and tissue samples were analysed by ELISA and real-time PCR. Neutrophils and hepatocytes were stimulated with cytokines and supernatant of* Staphylococcus aureus*. The present study demonstrates the induction and release of lysozyme in serum of multiple injured patients. The highest lysozyme expression of all tested cells and tissues was detected in neutrophils. Stimulation with trauma-related factors such as interleukin-6 and* S. aureus* induced lysozyme expression. Liver tissue samples of patients without trauma show little lysozyme expression compared to neutrophils. After stimulation with bacterial fragments, lysozyme expression of hepatocytes is upregulated significantly. Toll-like receptor 2, a classic receptor of Gram-positive bacterial protein, was detected as a possible target for lysozyme induction.

## 1. Introduction

Multiple trauma is the third most abundant cause of death for all ages, following heart disease and cancer. Generally multiple trauma results in a complex pathophysiological and immunological response. Up to now the molecular mechanisms are not fully understood. Various immunological alterations known as posttraumatic inflammatory responses depend on the individual injury pattern and the time elapsed after injury [1]. During the posttraumatic inflammatory reaction multiple cytokines are released by various cells [2]. IL-6 has recently proven to be of outstanding clinical interest and can now be used as a prognostic clinical marker in sepsis [3]. With respect to open wounds and fractures after multiple trauma, one could regard the overall infection rate as remarkably low [4]. It seems that there are mechanisms in the immune system preventing trauma patients from infections.

Several defense mechanisms are activated during the inflammatory reaction. Antimicrobial peptides (AMP) belong to the host defense peptides (HDP) and are key elements of the innate immunity, providing the first barrier in many epithelial cells against invading microbes. Although open fractures as well as wounds can give rise to local infections and sepsis, AMP regulation after trauma has barely been examined at all.

In previous investigations we verified that serum of multiply injured patients has a significantly higher antimicrobial capacity than serum of healthy donors. Further observations supported the hypothesis that small HDP were responsible for the antimicrobial effect. As possible mediators we detected an induction of the human beta-defensins 2 and 3 (hBD-2 and hBD-3) and the cathelicidin LL-37 in serum of multiply injured patients [5]. Although these HDP were significantly upregulated after trauma, the source of the high antimicrobial capacity of trauma serum could not be clarified completely.

Lysozyme is the first described host defense peptide [6] and operates synergistically with hBD-2, hBD-3, and LL-37 [7]. It is a key component of the innate immune system and kills bacteria by catalytic hydrolysis of cell wall peptidoglycan, but it also exhibits catalysis-independent antimicrobial properties [8, 9]. Belonging to host defense peptides, it also controls host physiological functions such as inflammation, angiogenesis, and wound healing [10]. Additionally the evaluation of lysozyme blood levels has clinical significance in the diagnostic of chronic diseases like sarcoidosis, rheumatoid arthritis, or Crohn's disease.

Nevertheless host defense peptide expression, especially of lysozyme, has not been fully elucidated yet with regard to inflammatory reaction.

In this study we analyzed serum of trauma patients for lysozyme induction with regard to the antimicrobial effect and investigated possible cellular sources. We examined possible stimulators and stimulation pathways for the expression of lysozyme.

## 2. Methods

### 2.1. Patient Population

32 adult patients with multiple injuries (aged 16–65 years) were prospectively studied. Inclusion criteria were an injury severity score (ISS) of 16 points or greater without the presence of severe traumatic brain injury (though abbreviated injury scales for head injury were included in the ISS) and primary admission to our institution. AIS for head injuries with a score of three or more were considered to be severe traumatic brain injury. Exclusion criteria were penetrating trauma, severe traumatic brain injury, and patients not directly admitted to our institution following the traumatic event. All procedures were in concordance with the Revised Version of the Declaration of Helsinki (41st general meeting in 1989 in Hong Kong). The local ethics committee approval was obtained for the study. First-degree relatives signed the informed consent for inclusion of the patient in the study, as the patients were intubated and anaesthetized.

### 2.2. Patients Management and Treatment

A standardized clinical examination was followed by a focused sonography of the abdomen and pelvis. All patients received a trauma CT-scan of head, chest, abdomen, pelvis, and spine. The results were analyzed by a radiologist and an attending trauma surgeon. At the time of admission to the intensive care unit (ICU), the clinical examination and the focused assessment including sonography were repeated. Fractures were stabilized as soon as possible either by definitive internal fixation or by temporary external stabilization. If the patient required laparotomy, stabilization of fractures was performed afterwards.

### 2.3. Control Group

A group of 6 healthy volunteers (3 women and 3 men) at the age of 18 to 65 years were randomly selected for determination of the normal baseline values of all parameters evaluated. Volunteers had not previously suffered any trauma or acute or chronic medical problems.

### 2.4. Collection of Blood

Venous EDTA-blood (9 mL Monovette, Sarstedt, Germany) was collected daily at 7 a.m. from traumatized patients over a 14-day period for plasma preparation. The first sample was drawn directly after admission to the hospital. The blood was centrifuged for 10 min at 2000 g at room temperature within 30 minutes. The supernatant serum was stored at −80°C.

### 2.5. Lysozyme ELISA

Lysozyme ELISA was performed as described by Varoga et al. [11]. The lysozyme capture antibody and detection antibody were provided by Nordic Immunological Lab, The Netherlands.

### 2.6. Real-Time PCR

For real-time polymerase chain reaction (PCR), RNA was isolated from the cultured human hepatocytes (HepG2) with the RNeasy-Total RNA Kit (Qiagen, Hilden, Germany) according to the manufacturer's instructions. RNA purification of whole blood samples of multiply injured patients and healthy volunteers was performed with QIAamp RNA Blood Mini Kit (Qiagen, Hilden, Germany). Total RNA (1,000 ng) was used for reverse transcription and subsequent real-time PCR with gene-specific primers. Real-time PCR was performed as described by Varoga et al. [11]. The gene of interest was detected using TaqMan probes: Hs01548808_m1 (Applied Biosystems, Foster City, CA, USA).

### 2.7. Recruitment of Tissues

Epidermis was collected from patients undergoing osteosynthesis after fracture. After skin incision a 50 × 2 mm piece of epidermis was cut for further examinations. Liver tissue was harvested from patients undergoing liver resection. 10 mg of macroscopic healthy tissue was cut for further examination. All operations were performed at the Departments for Trauma Surgery and Visceral Surgery at the University Medical Center of Schleswig-Holstein, Kiel Campus. The local ethics committee approval was obtained for the study.

### 2.8. Isolation of PMN with Polymorph-Prep^©^

Venous blood samples of 9 mL were taken from healthy volunteers by the use of EDTA Monovette. PMN were isolated from whole blood using Polymorph-Prep^©^ (Axis-Shield, Norway) according to the manufacturer's instructions. 5 mL of blood was carefully layered on 5 mL of Polymorph-Prep^©^. After centrifuging at 500 ×g for 30 min at room temperature mononuclear cells and polymorphonuclear cells were separated and resuspended in 0.45% NaCl.

### 2.9. Cultivation and Stimulation of PMN

For experiments isolated cells were ingested in RPMI 1640-cell culture medium with 7% fetal bovine serum, 1% L-glutamine (2 mM), and 1% penicillin/streptomycin (5000 U/mL/5000 *μ*g/mL). PMN were applied on 6-well plates with 1.5*·*10^6^ cells in each well and stimulated with 50 ng/mL interleukin (IL-6) (Tebu, Offenbach, Germany) and SA (supernatant 1 : 100; assembled as described by Gläser et al. [12]) at 37°C for 24 h. Concentration of lysozyme in supernatant was measured by ELISA. Every experiment was conducted three times and results were compared with unstimulated cells.

### 2.10. Isolation of Monocytes

Venous blood samples of 9 mL were taken from healthy volunteers by the use of EDTA Monovette. Mononuclear cells located in the interphase after use of Polymorph-Prep were ingested in 30 mL iced phosphate buffer saline and washed 5 times with 30 mL phosphate buffer saline at 300 ×g. Mononuclear cells were ingested in iced medium free of serum and counted in a counting chamber for adjustment of cell concentration of 10^7^ cells/mL. Meanwhile autologous serum gained from density gradient centrifugation was centrifuged, respectively, at 720 ×g and 1600 ×g. Petri dishes were incubated with serum-free thrombocytes for 15 min. After washing the petri dishes with 5 mL phosphate buffer, saline fetal calf serum was added to mononuclear cells so that a concentration of 3% fetal calf serum in medium was generated. All the dishes were filled with 5 mL of cell suspension and incubated for 45 min under standard conditions (37°C, 5% CO_2_, 21% O_2_, and 98% humidity). Accordingly nonadherent cells were rinsed with warm phosphate buffer saline. Dishes with remaining adherent cells were covered with 5 mL iced serum-free medium and incubated on ice for 1 h. After 1 h a sphering of monocytes caused by cold stimulus could be seen through a transmitted light microscope so that these cells could be rinsed with iced phosphate buffer saline by the use of a 1000 *μ*L pipette. Monocytes were collected in a 10 mL tube and washed in iced phosphate buffer saline at 600 ×g. The pellet was resuspended in RPMI medium. Cells were counted by the use of a counting chamber and carefully examined for vitality by suspension in trypan blue. Vitality was constantly more than 95%.

### 2.11. Cultivation and Stimulation of Hepatocytes

The experiments were performed with immortalized liver cells, HepG2 (Cell Lines Service, Germany). These cells represent histological and biological characteristics of differentiated parenchymal liver cells [13].

Cells were cultivated as described in distributer's instructions. For cultivation 800,000 cells were filled into a 25 cm^2^ cell culture bottle with 2 mL of cell medium. Cells were stimulated with 50 ng/mL IL-6 and 1 : 100 supernatant of SA. For blocking experiments, cells were incubated with 10 *μ*g/mL TLR2 antibody (eBioscience, San Diego, USA) for 1 h and stimulated afterwards. Incubation was performed at 37°C in ambient humidity with 5% CO_2_ in an incubator. Every experiment was accomplished three times and results were compared with unstimulated cells.

### 2.12. Statistical Analysis

Data is expressed as the mean ± SD of tested samples. Statistical significance was evaluated using one-way ANOVA with Bonferroni correction. Statistical significance was assumed where probability values of less than 0.05 were obtained. Spearman's linear regression analysis was performed to evaluate correlation of protein serum concentrations.

## 3. Results

### 3.1. Elevated Lysozyme Levels in Serum of Multiply Injured Patients

**Figure 1:**
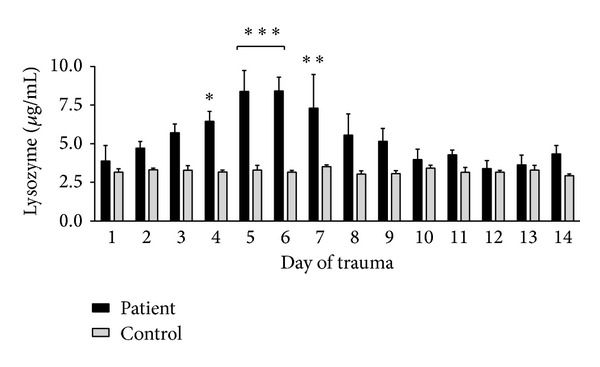
*Elevated lysozyme levels in serum of multiply injured patients*. Time course of serum concentration of lysozyme over a period of 14 days detected by ELISA. Significant elevated concentration of lysozyme can be shown between 4 and 7 days after trauma as compared to healthy controls; ***P < 0.001, **P < 0.01, and *P < 0.5.

To investigate whether lysozyme is induced in serum after trauma, we analyzed sera of trauma patients (ISS > 16) over a period of 14 days and compared them to a healthy control group. Lysozyme concentrations doubled within the first week and decreased afterwards (Figure 1). Maximum levels were measured on day 6 (6.5 ± 2.0 *μ*g/mL). Significant elevations of lysozyme concentrations could be demonstrated between day four and day seven in comparison to the healthy control group (3.15 ± 0.1 *μ*g/mL).

### 3.2. Induction of Lysozyme Expression in Leukocytes of Multiply Injured Patients

**Figure 2:**
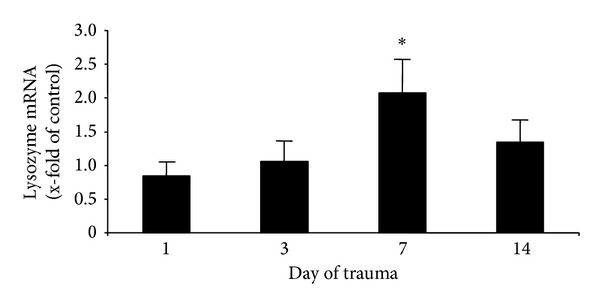
*Induction of lysozyme expression in leucocytes of multiply injured patients*. Gene expression analysis using real-time PCR in leukocytes of multiply injured patient indicates on day 7 significant higher lysozyme levels as compared to healthy controls; *P < 0.01.

After detecting lysozyme in serum of multiply injured patients, we tried to identify several sources for this peptide. An upregulation of host defense peptides after infection has been shown before for skin, liver, and blood cells, especially for monocytes and polymorphonuclear leukocytes (PMN) [14]. Therefore we focus on these cell lines in the present study. As a possible source for serum levels, we analyzed leukocytes of whole blood drawn from multiply injured patients. Corroborating the protein levels in serum, real-time PCR revealed a significant increase in lysozyme gene expression on day 7 (2.1 ± 0.4 compared to control (Figure 2)).

### 3.3. Lysozyme Expression in PMN and Monocytes

**Figure 3:**
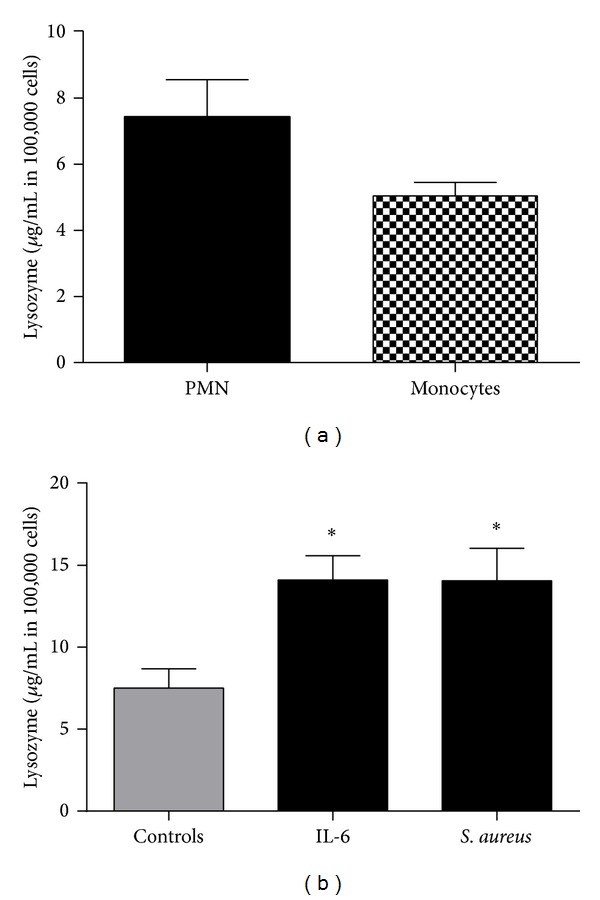
*Lysozyme expression in PMN and monocytes*. (a) Polymorphonuclear cells (PMN) and monocytes were isolated from whole blood of healthy donors and lysed cells were detected for lysozyme concentration by ELISA. PMN contain higher levels compared to monocytes. (b) PMN were incubated with 50 ng/ml IL-6 and SA (supernatant 1 : 100); *P < 0.01.

A great amount of HDP is stored in granules of PMN and makes up to 50% of their granule content. We tested PMN and monocytes as important cellular mediators of posttraumatic inflammatory serum response examining whether they express comparable concentrations of lysozyme (Figure 3(a)). PMN contained a higher concentration of lysozyme (7.4 ± 1.1 *μ*g/mL in 100,000 cells) than monocytes (5.1 ± 0.7 *μ*g/mL in 100,000 cells).

### 3.4. In Vitro Stimulation of PMN

IL-6 is a well-known cytokine, which is upregulated after multiple trauma and correlates with the risk of multiple organ dysfunction syndrome (MODS) after multiple trauma [15]. Major causes for hyperinflammation and systemic inflammatory response syndrome (SIRS) which may lead to MODS are invading* Staphylococcus aureus* (*S. aureus*) in the context of extensive soft tissue damage. To examine whether lysozyme is induced by typical stimulants of posttraumatic inflammation, PMN were incubated with IL-6 and* S. aureus*. Both stimulants increased the concentrations of lysozyme significantly as shown in Figure 3(b): IL-6 (14.1 ± 1.5 *μ*g/mL in 100,000 cells),* S. aureus* (14.1 ± 1.9 *μ*g/mL in 100,000 cells).

### 3.5. Lysozyme Expression in PMN, Liver, and Epidermis

**Figure 4:**
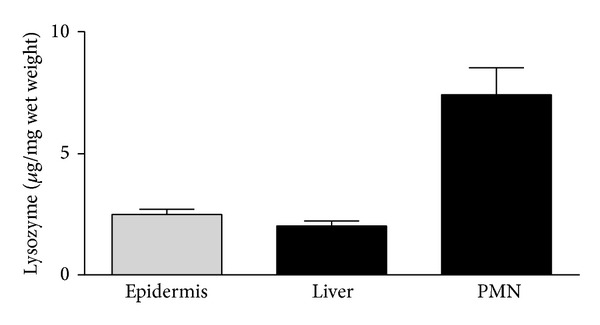
*Source of lysozyme in different tissues*. Lysozyme levels in tissue of healthy donors were analyzed by ELISA. Highest lysozyme concentration was detected in neutrophils, whereas liver and epidermis showed nearly similar expressions.

With regard to further sources of lysozyme expression, we analyzed samples of liver without severe injuries and compared them to epidermis, a well investigated source of lysozyme. In relation to the wet weight, the highest amount of intracellularly stored lysozyme was measured in PMN (7.4 ± 1.1 *μ*g/mg wet weight). Samples of liver contained similar amounts of lysozyme as epidermis samples (2.0 ± 0.2 *μ*g/mg and 2.5 ± 0.2 *μ*g/mg wet weight) (Figure 4).

### 3.6. Induction of Lysozyme in Cultured Hepatocytes

**Figure 5:**
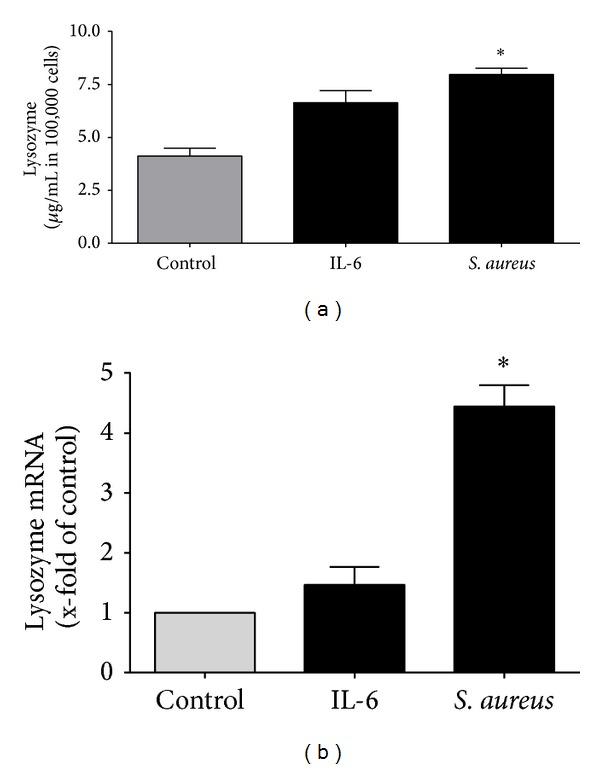
*Induction of lysozyme in hepatocytes*. (a) HepG2 cells were incubated with IL-6 and* S. aureus* for 24 hours. Significant increase of lysozyme concentration was detected by ELISA after stimulation with* S. aureus*. (b) To evaluate transcriptional induction of lysozyme, cultivated hepatocytes (HepG2) were stimulated with IL-6 and* S. aureus*. Gene expression of lysozyme was measured by real-time PCR after stimulation; *P < 0.01.

The high amounts of lysozyme in liver tissue led us to the idea to further analyze the induction. Liver and especially hepatocytes play an important role in peptide expression in posttraumatic inflammation [16]. We cultivated immortalized hepatocytes (HepG2) and stimulated them with IL-6 and* S. aureus* (Figure 5(a)). Lysozyme expression was increased after stimulation with both stimulants but only stimulation with* S. aureus* (8.0 *μ*g/mL in 100,000 cells ± 0.3) revealed a significant increase compared to controls (4.1 *μ*g/mL in 100,000 cells ± 0.4). Possible transcriptional induction of lysozyme was tested by real-time PCR. As expected from protein assay, both enhance gene expression of lysozyme in HepG2, but only* S. aureus* showed significant induction compared to control (Figure 5(b)).

### 3.7. TLR2, a Pathway for Lysozyme Induction

**Figure 6:**
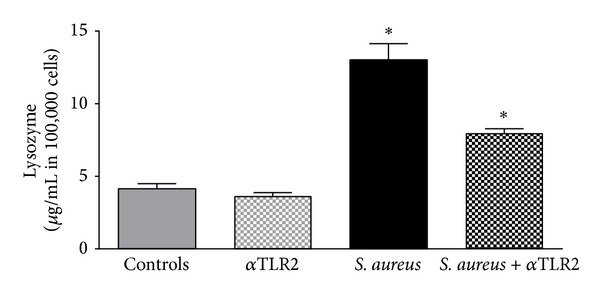
*TLR2, a pathway for lysozyme induction*. The induction of lysozyme in HepG2 cells was measured by ELISA after blocking TLR2 without stimulation, after stimulation with* S. aureus*, and after stimulation with* S. aureus* and additional blocking of TLR2. Stimulation with* S. aureus* and stimulation with* S. aureus* after blocking of TLR2 showed significant increase.

To examine whether TLR2 plays a role in lysozyme expression, we stimulated HepG2 cells with* S. aureus* and in combination with the blocking antibody for TLR2 (Figure 6). Treatment of HepG2 cells with TLR2 blocking antibody alone did not lead to an alteration of lysozyme expression. Stimulation of HepG2 cells with* S. aureus* induced an upregulation of lysozyme concentration (8.0 *μ*g/100 000 cells ± 1.4). Compared to stimulation of HepG2 cells with* S. aureus* alone, stimulation with* S. aureus* in combination with TLR2 blocking antibody showed a significant decrease of lysozyme concentration (5.9 *μ*g/100 000 cells ± 1.4), suggesting a major role of TLR2 in the regulatory process.

## 4. Discussions

AMP are effector molecules of the innate immunity and act as endogenous antibiotics by directly killing bacteria, virus, and fungi. Due to rising numbers of multiresistant infections, research on these peptides has increased rapidly during the last decade. Particularly multiply injured patients with a combination of wounds and open fractures are faced by a multiplicity of invading Gram-positive and Gram-negative bacteria. In previous investigations we showed that serum of trauma patients reveals an enhanced antimicrobial effect in comparison to healthy volunteers serum [5]. In this study we documented a significant induction of lysozyme in serum of multiply injured patients. In contrast to hBD-2 and hBD-3, lysozyme expression was upregulated up to 8 times compared to its minimal effective dose of antibacterial protein expression in serum (1 *μ*g/mL) [8]. Additionally it is known to act synergistically with further AMP like hBD-3, which is also induced in trauma serum [5, 7]. Lysozyme cracks the polysaccharide chain of the bacteria wall and therefore hBD can infiltrate easily [17].

To extend the comprehension of lysozyme regulation in patient's serum, we investigated possible sources of lysozyme. Lysozyme is constitutively expressed by inflammatory cells like monocytes and PMN [18]. Particularly neutrophils play an important role in the defense of the injured tissue from the first 10 minutes until 3 days after trauma. Inflammatory mediators like host defense peptides are induced all along [19, 20]. Severe injury generates activation of different inflammatory cells such as granulocytes, monocytes, and macrophages [21] whereas especially monocytes and neutrophils are well known for lysozyme expression [22]. Lysozyme is stored in granules of human neutrophils [23] that are activated immediately after trauma [24]. In this study we were able to document the induction of lysozyme in leucocytes of multiple trauma patients with comparable progression to the serum concentration. Lysozyme induction in different cell lines has been reported for* S. aureus* and numerous cytokines like IL-6, IFN-*γ*, and IL-17 [16, 25, 26]. Particularly IL-6 plays a major role in posttraumatic inflammatory reaction [27]; therefore, we tested the key player for its effect on lysozyme expression in PMN. As shown before bacteria seem to be a strong inducer for lysozyme. With regard to open fracture, wounds, and deep tissue contusion, bacteria constitute a major cause for systemic infection after trauma. Our in vitro data revealed that both IL-6 and* S. aureus* induce lysozyme in PMN.

Relating to systemic inflammation and shock syndrome, liver constitutes a key figure in terms of the regulation of mediators. Markers of acute-phase reactants are usually of hepatic origin, are activated by cytokines [28], and regulate innate defense after severe injury [29]. Interestingly we detected the same amounts of lysozyme in liver tissue and in skin, which is known for huge lysozyme expression [30]. Therefore we investigated hepatocytes for regulation of lysozyme and ascertained upregulation provoked by IL-6 and Gram-positive bacteria. In multiply injured patients Kupffer cells remove pathogens which invaded in case of open wounds through phagocytosis. The role of lysozyme in killing bacteria in these cells has not fully been elucidated yet. Additionally the liver resembles a central organ of cytokine activity; particularly Kupffer cells of the liver release a variety of cytokines. These cytokines might stimulate hepatocytes to express lysozyme.

To get one step forward to comprehend the regulation of lysozyme, we identified one signaling pathway in hepatocytes. TLR2 plays a major role in the induction of AMP [31]. As shown before, induction of AMP is regulated by Toll-like receptors [31, 32] which are activated by cytokines and bacterial fragments [25, 32]. These receptors are associated with the induction of nuclear factor-kappa B (NF-*κ*B) and the induction of antimicrobial peptides [33]. NF-*κ*B was defined to bind to NF-*κ*B-like sequence of the lysozyme promoter and induce lysozyme expression in macrophages and PMN [34]. Therefore we focused on the lysozyme induction in hepatocytes in the present study and were able to document that the blockade of TLR2 in stimulated hepatocytes showed a downregulating effect on lysozyme expression. We assume that TLR2 is involved in lysozyme regulation not only in PMN, but also in hepatocytes.

## 5. Conclusion

With this study we showed high antimicrobial activity and significant upregulation of lysozyme induced by trauma relevant mediators for the first time. With regard to the application of AMP in context of systemic infection after multiple trauma, these results bring us one step closer to the comprehension of the immune system of multiply injured patients. Considering the complexity of this issue, lysozyme will require further attention.
